# Supplementary material for: An Intelligent Compaction Analyzer: A Versatile Platform for Real-Time Recording, Monitoring, and Analyzing of Road Material Compaction
Source: Sensors (Basel). 2023 Aug 29;23(17):7507. doi: 10.3390/s23177507 (PMC10490652; doi:10.3390/s23177507)
Supplement: Supplementary file 1 [file sensors-23-07507-s001.zip › sensors-2487777-supplementary.pdf]

Table S1. Abbreviation and acronyms.

Acronym	Meaning
CCV	Compaction Control Value
CMV	Compaction Meter Value
CPU	Central Processing Unit
DAQ	Data Acquisition
DFT	Discrete Fourier Transform
GPR	Ground Penetrating Radar
GPU	Graphics Processing Unit
GNSS	Global Navigation Satellite System
HDMI	High-Definition Multimedia Interface
HPA	Horizontal Position Accuracy
IEPE	Integrated Electronics Piezo-Electric
IC	Intelligent Compaction
ICA	Intelligent Compaction Analyzer
ICM	Intelligent Compaction Mete
ICMV	Intelligent Compaction Measurement Values
IR	Infrared
SK_b	Stiffness
LGPL	LESSER GENERAL PUBLIC LICENSE
MFD	Modified Fundamental Distortion
NTRIP	Networked Transport of RTCM via Internet Protocol
PCB	Printed Circuit Board
PPM	Pixels Per Meter
RAM	Random Access Memory
ROI	Region of Interest
RTK	Real-Time Kinematic
RTCM	Radio Technical Commission for Maritime
SBC	Single Board Computer
SE_{vib}	SPARC Vibratory Modulus
SSID	Service Set Identifier
SSS	Solid-State Storage
THD	Total Harmonic Distortion
UI	User Interfaces
USB	Universal Serial Bus
VNC	Virtual Network Computing

Table S2. Software library dependencies.

Library	Description/Purpose	License
PySide2	GUI library used for interface development	Lesser General Public License (LGPL)
PyQtGraph	Graphics library used for developing visualization tools	MIT License (MIT)
Numpy	Array library used for 2D pixel array manipulation	BSD License (BSD)
Pillow	Image Manipulation	Historical Permission Notice and Disclaimer (HPND)
SciPy	Mathematical Functions library	BSD License (BSD)
Matplotlib	Visualization library used here for color maps	Python Software Foundation License (PSF)
pymea2	library for parse NMEA sentences	MIT License (MIT)
daqhats	drivers for MCC172 DAQ	NA
ads1015	Reading analog data	MIT License (MIT)
Pickle	Saving and opening byte data	Python Software Foundation License (PSF)

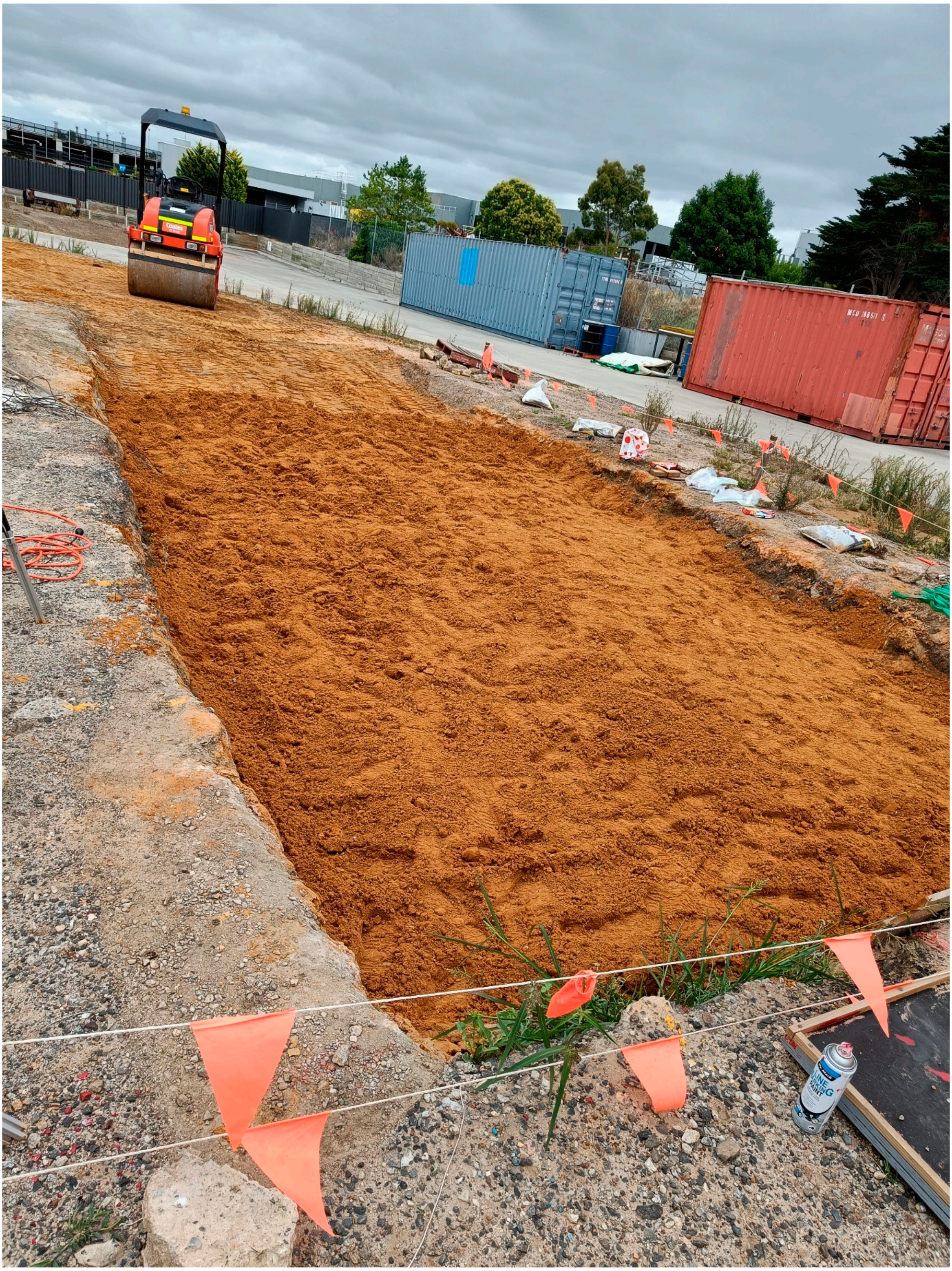

Figure S1. Preliminary experiments were carried out on an outdoor test pit for the ICA device.

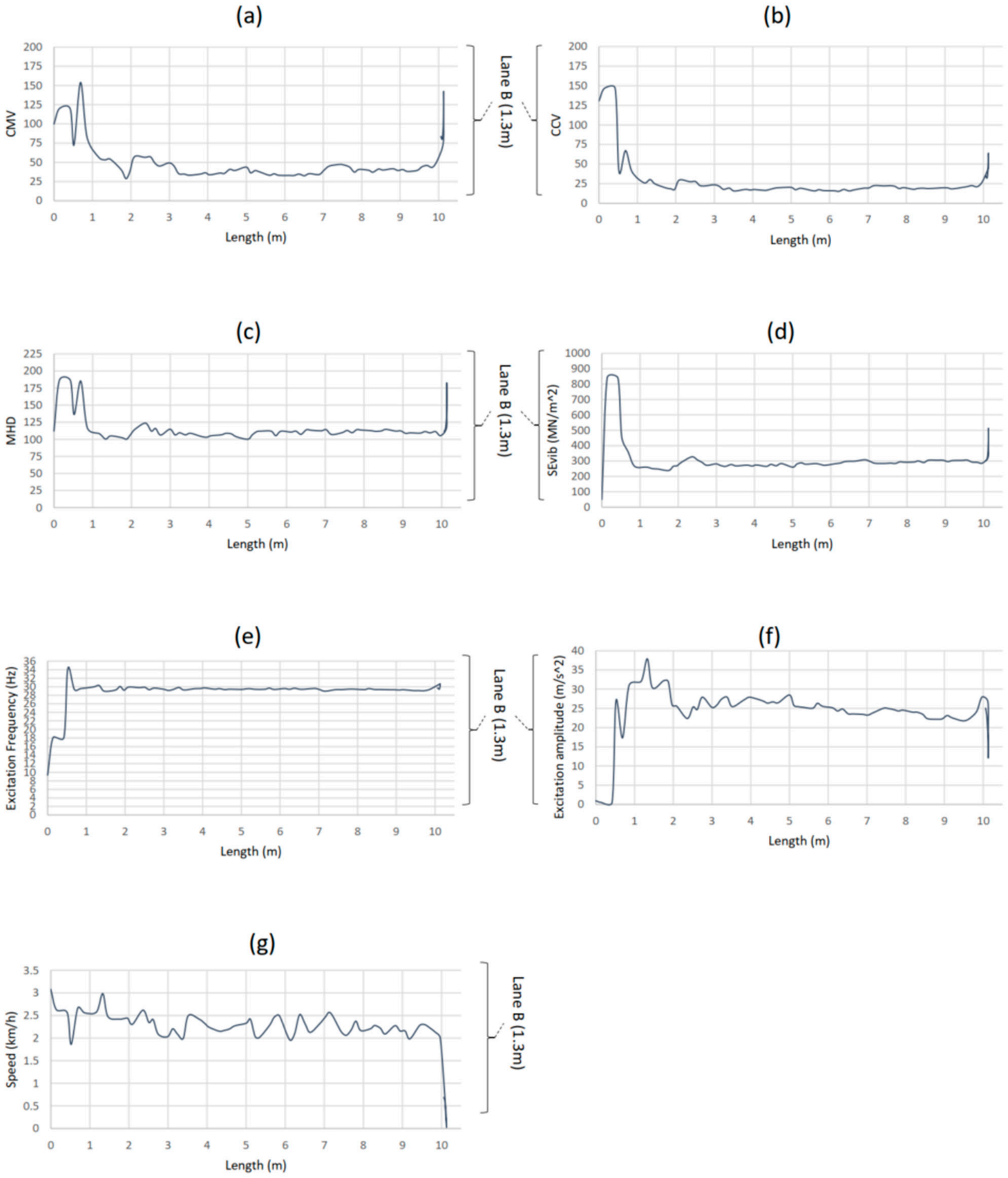

Figure S2. Different parameters of ICA device collected for a soil layer over 10m length. The peaks at the beginning and end refers the starting and stopping of the compaction roller.

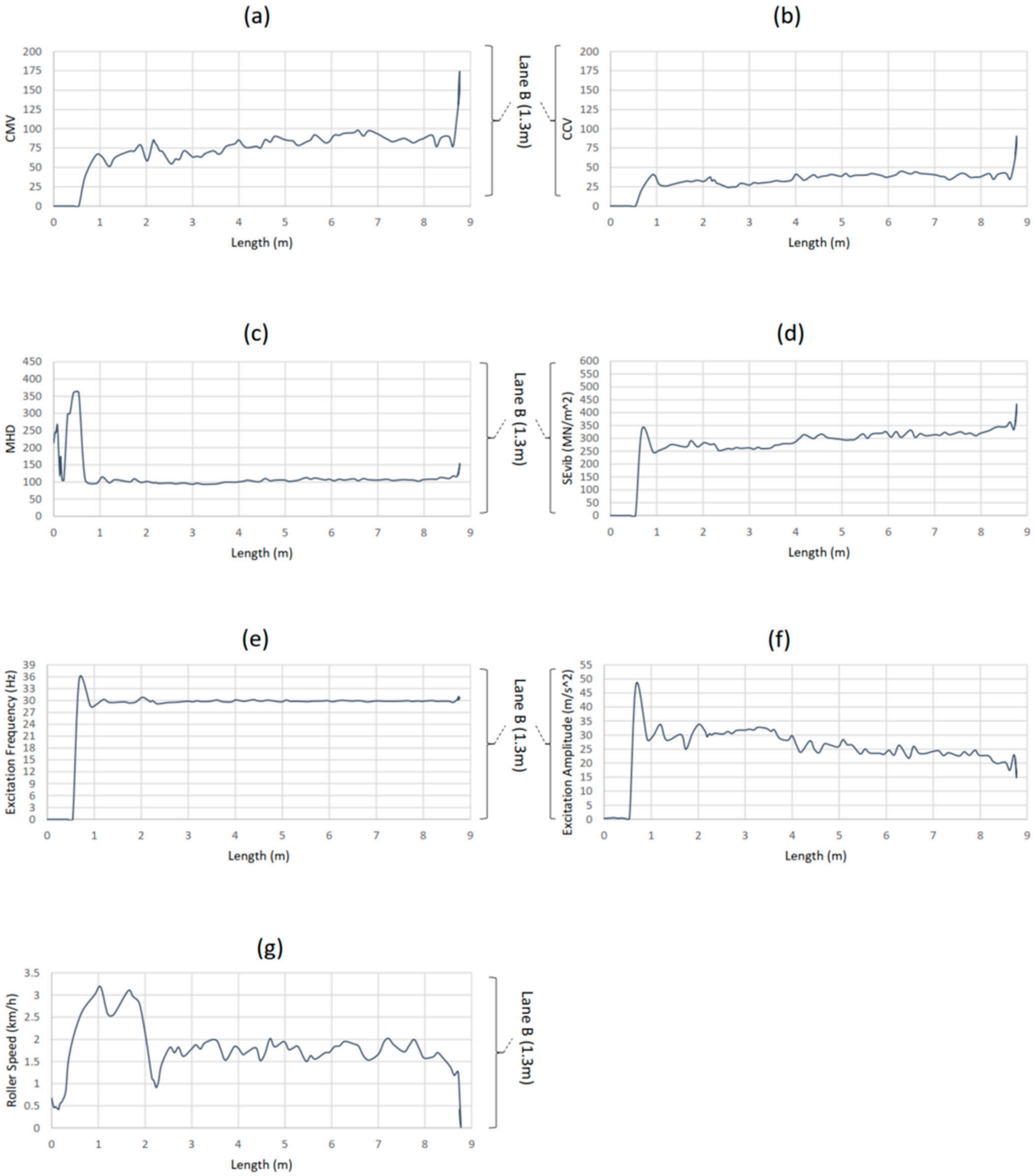

Figure S3. Different parameters of ICA device collected for a unbound granular material (UGM) layer over 10m length. The peaks at the beginning and end refers the starting and stopping of the compaction roller.

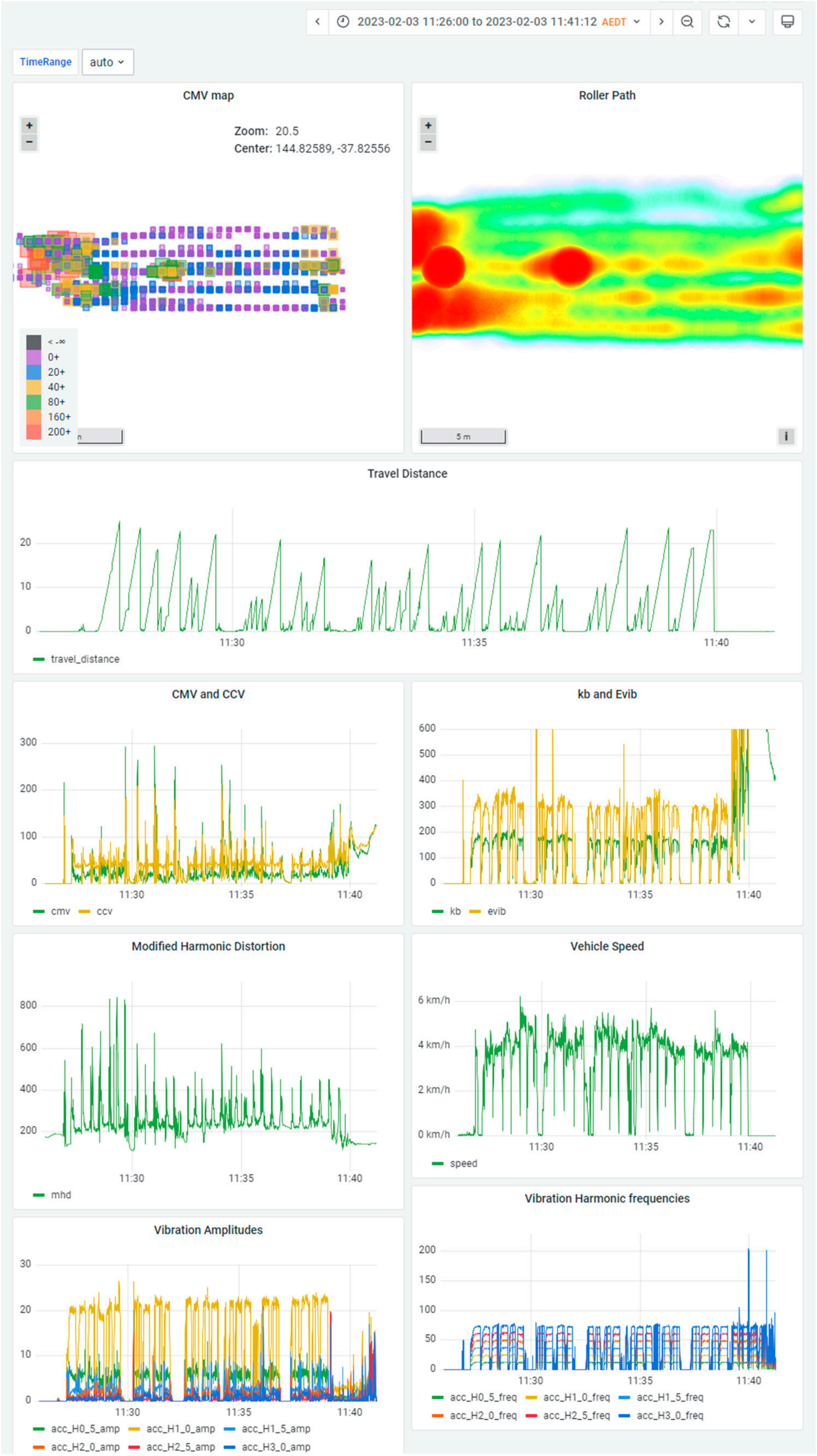

Figure S4. ICA platform web interface for data analysing powered by AWS Grafana.

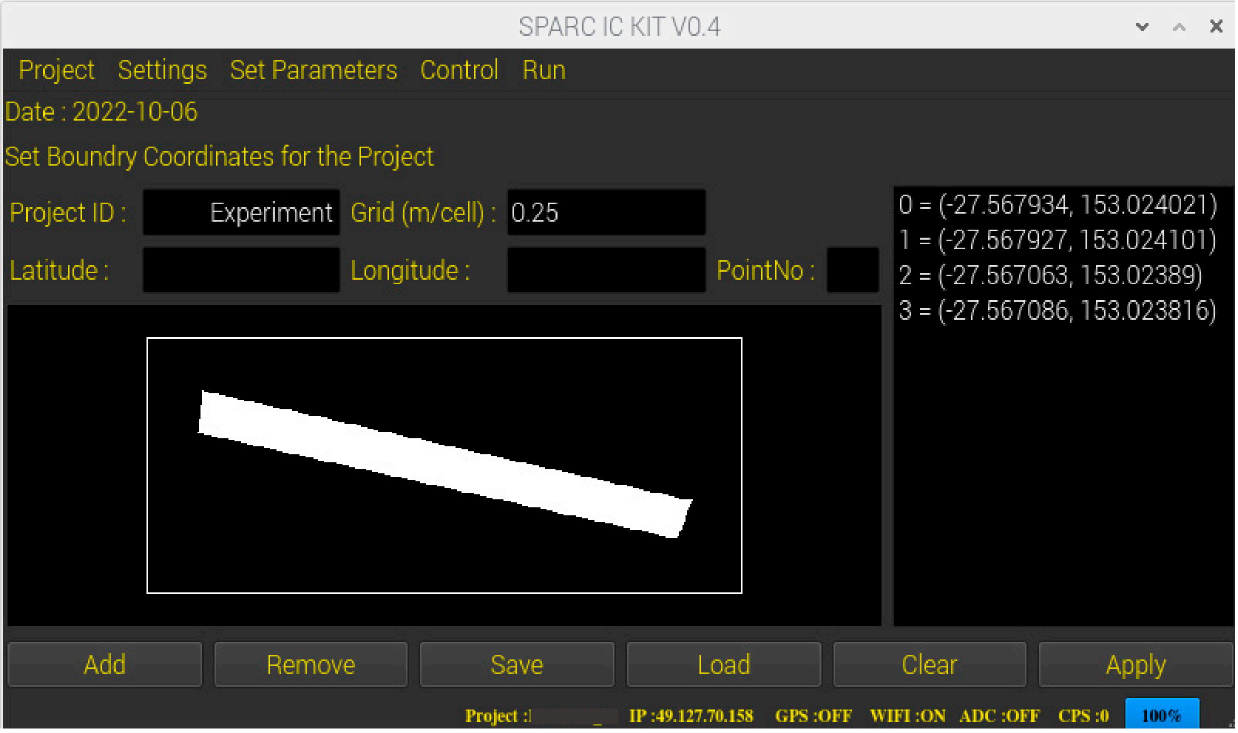

(a) Project User Interface

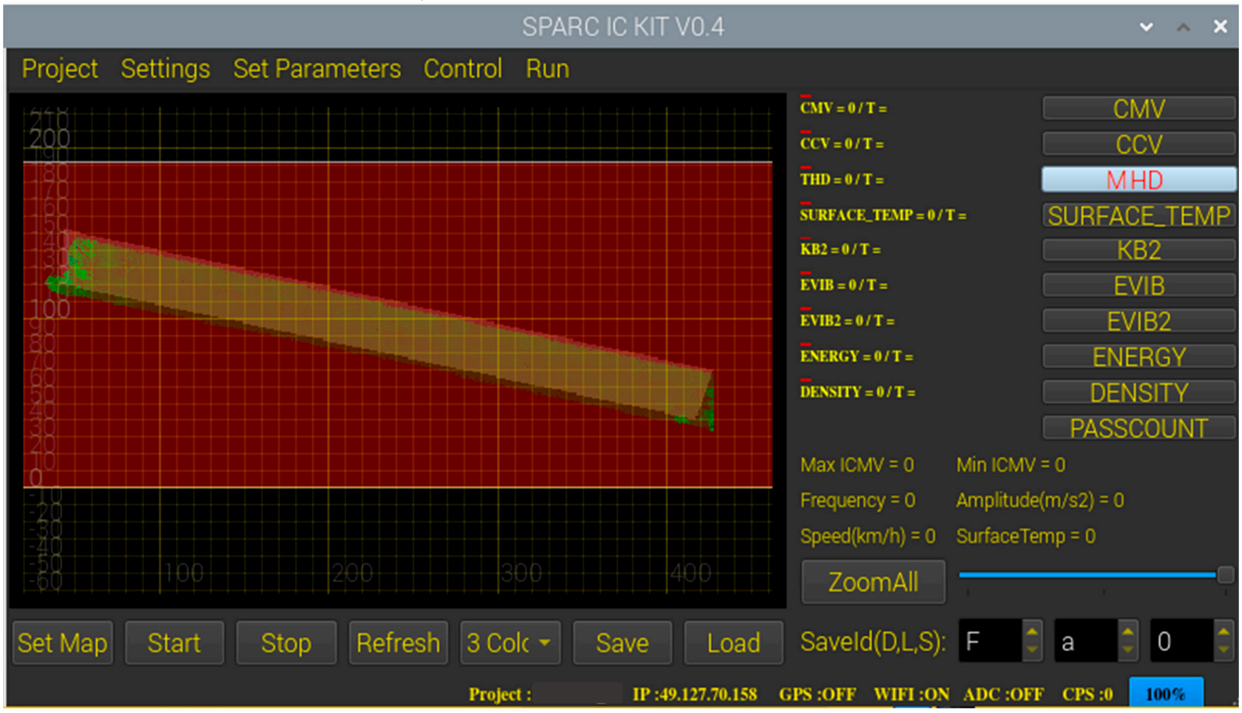

(b) Mapping User Interface

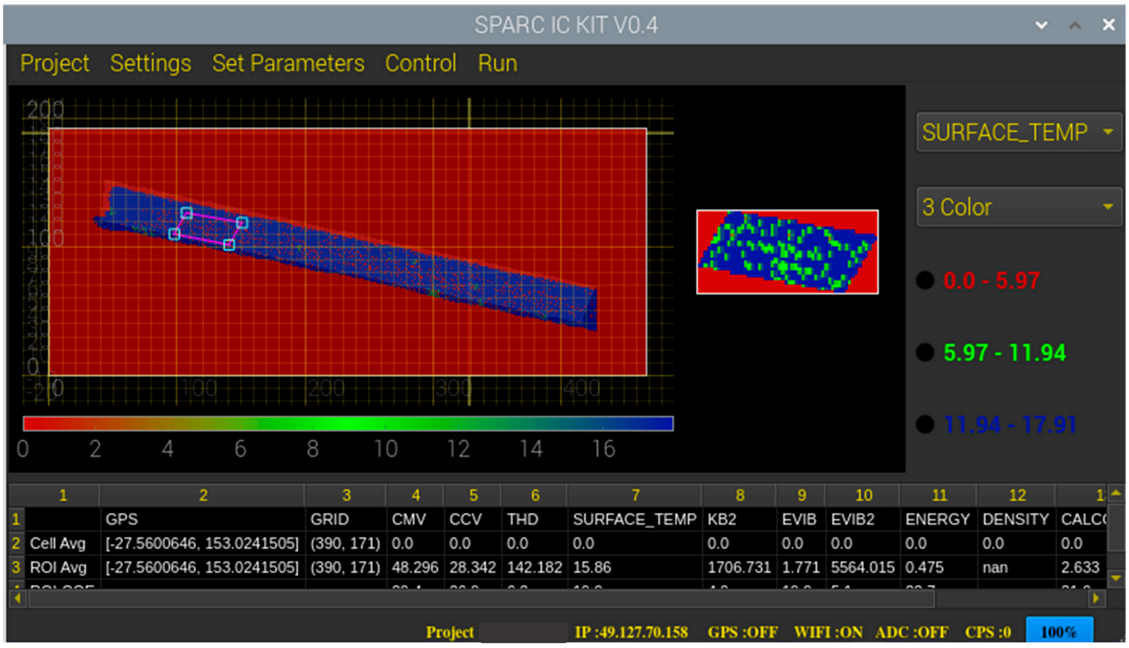

(c) Data Analyzing User Interface

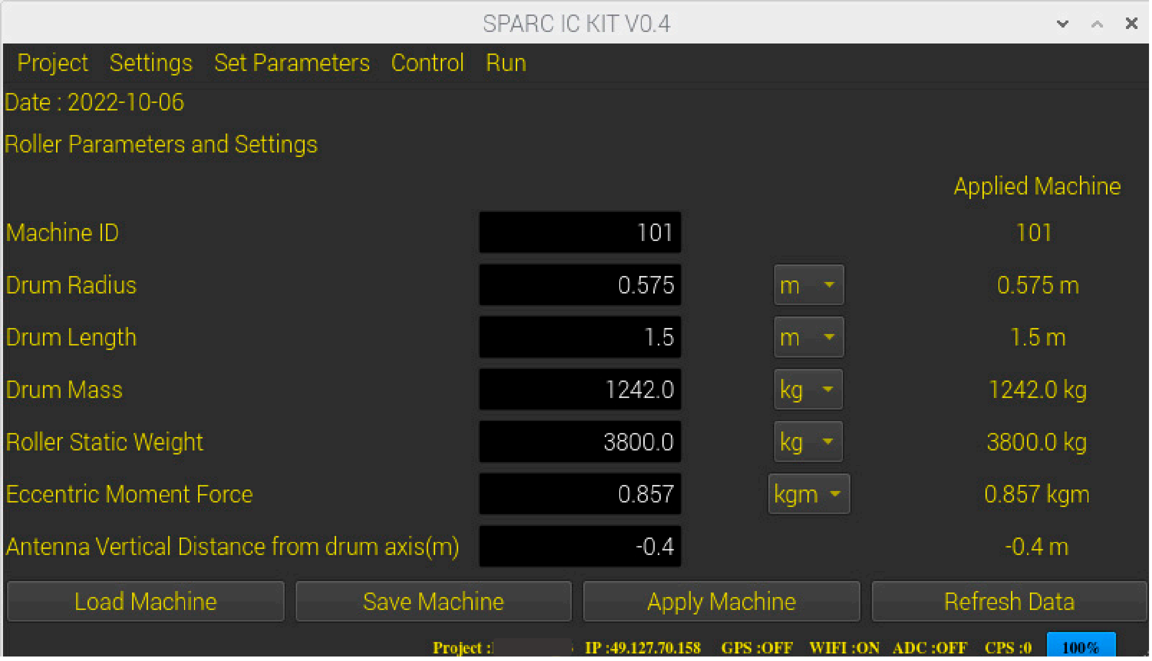

(d) Roller data User Interface

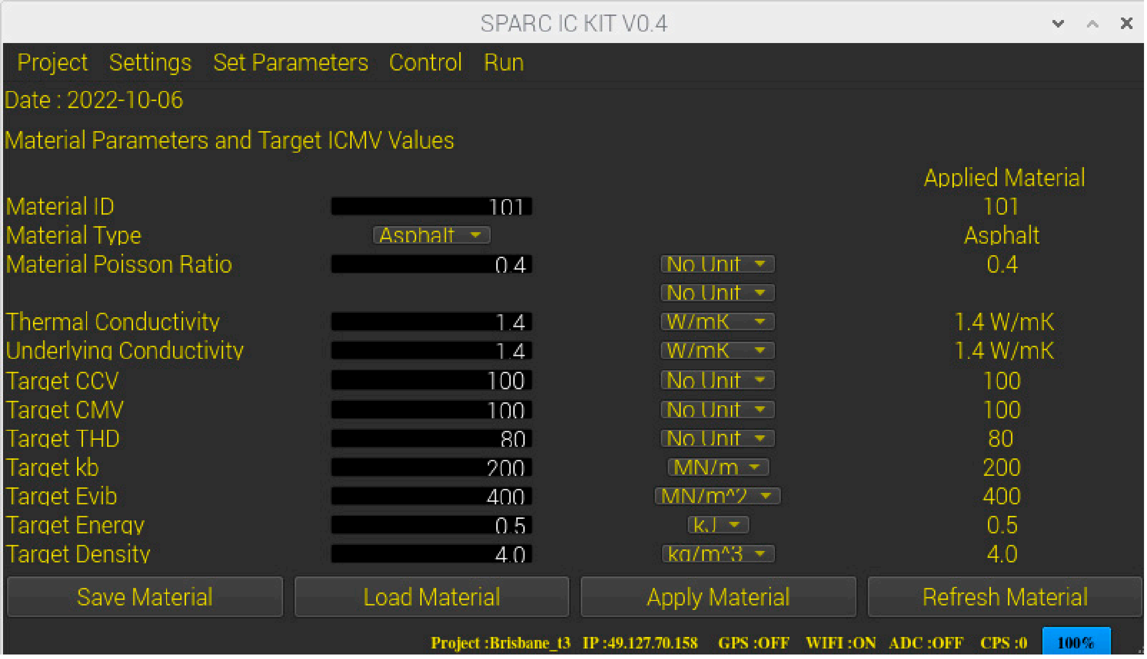

(e) Material data User Interface

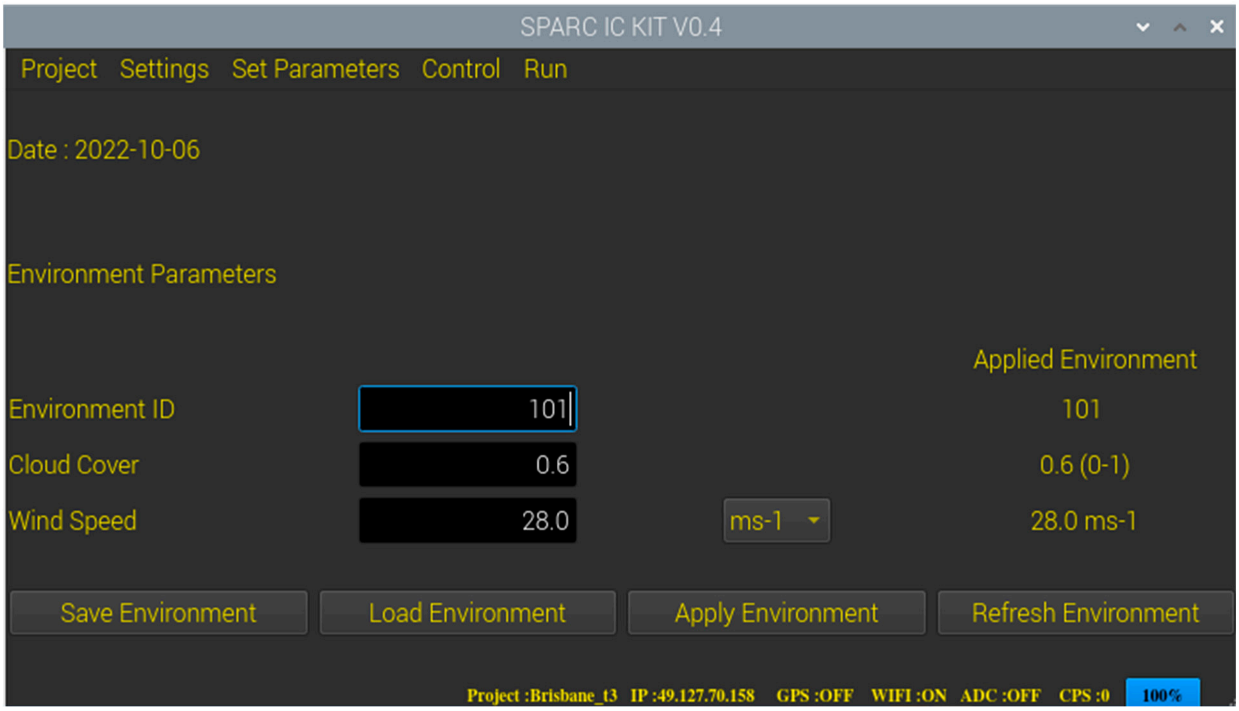

(f) Environment data User Interface

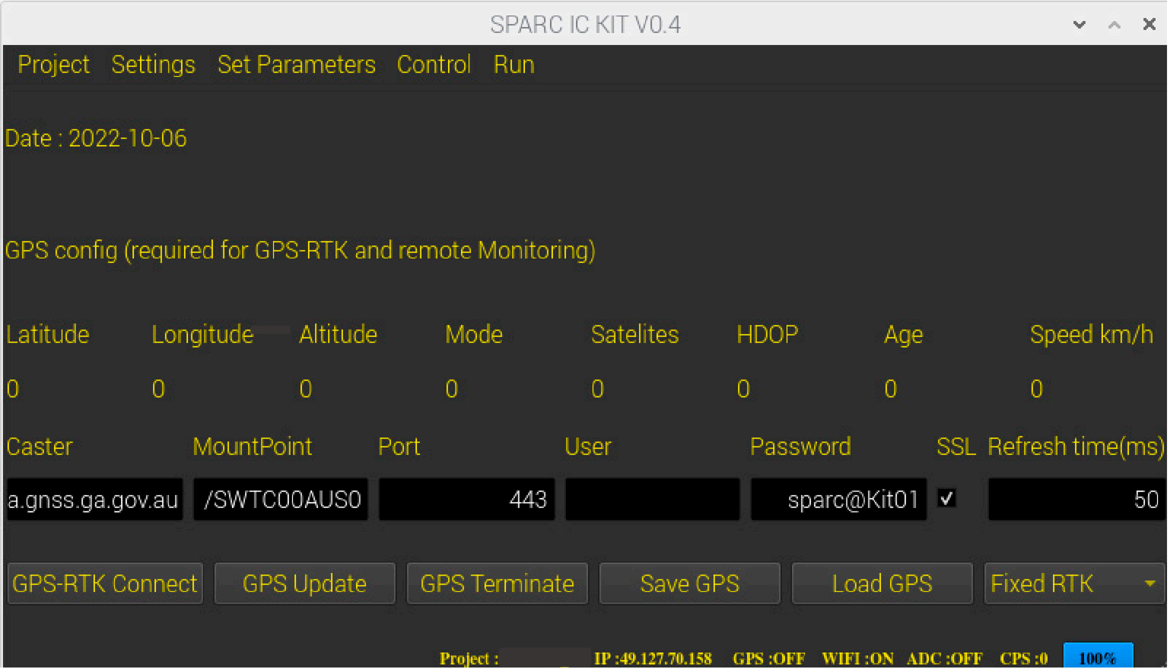

(g) GPS-RTK settings User Interface

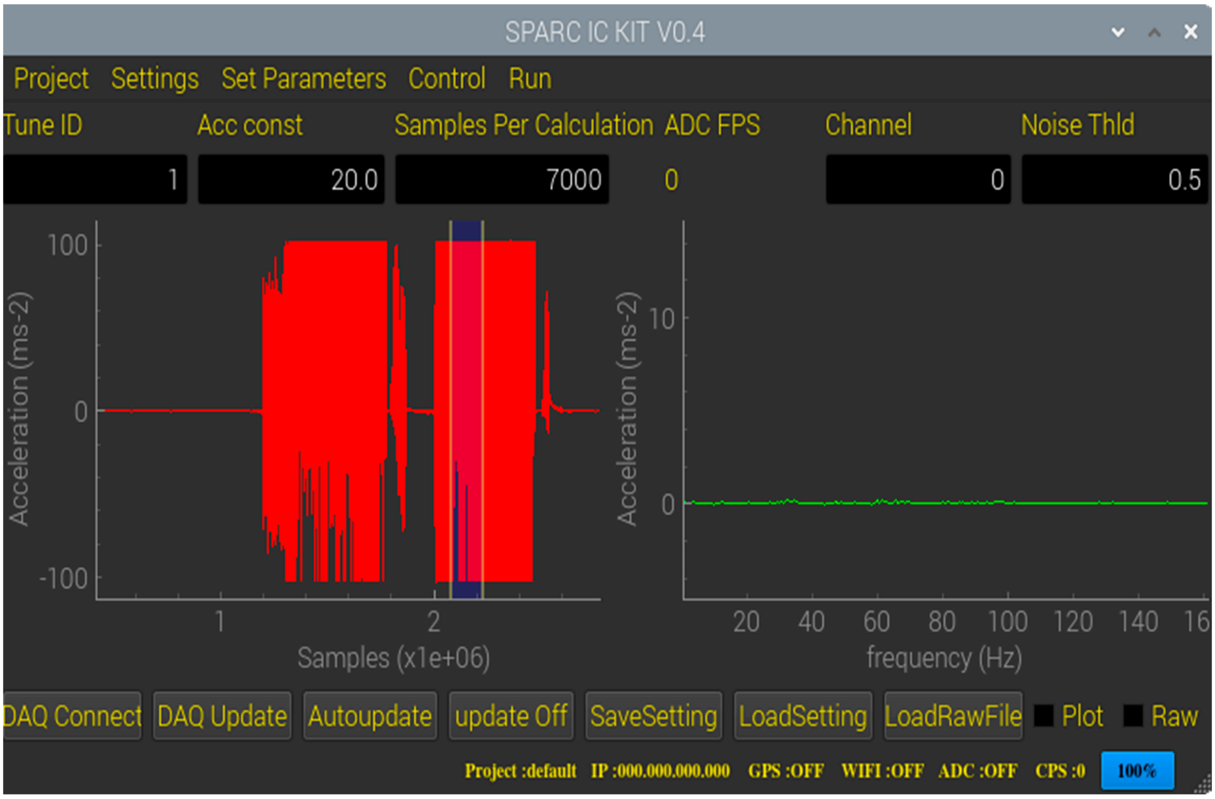

(h) DAQ data settings User Interface

Figure S5. All user interfaces (UI) of ICA device.
